# Paraskeletal and extramedullary plasmacytomas in multiple myeloma at diagnosis and at first relapse: 50-years of experience from an academic institution

**DOI:** 10.1038/s41408-022-00730-5

**Published:** 2022-09-16

**Authors:** Raquel Jiménez-Segura, Laura Rosiñol, Ma Teresa Cibeira, Carlos Fernández de Larrea, Natalia Tovar, Luis Gerardo Rodríguez-Lobato, Esther Bladé, David F. Moreno, Aina Oliver-Caldés, Joan Bladé

**Affiliations:** 1 Hematology department, Amyloidosis and Myeloma Unit, Hospital Clínic de Barcelona, Barcelona, Spain; 2 Institut d’Investigacions Biomèdiques August Pi i Sunyer (IDIBAPS), Barcelona, Spain; 3 University of Barcelona, Barcelona, Spain

**Keywords:** Myeloma, Myeloma

## Abstract

From January 1970 to December 2018, 1304 patients were diagnosed with multiple myeloma (MM) at our institution and 256 (19.6%) had plasmacytomas (Ps) (paraskeletal –PPs- 17.6%, extramedullary –EMPs-1.9%). Patients with Ps had lower serum M-protein and less advanced ISS stage than those without. At first relapse, 192 out of 967 patients (19.8%) developed Ps (PPs 14.6%, EMPs 5.1%). The only factor associated with Ps at relapse was the presence of Ps at diagnosis (46% vs 13%, *p* < 0.00001) with no impact with exposure to novel drugs or previous autologous stem-cell transplantation (ASCT). The median overall survival (OS) was 45, 44 and 20 months for patients without Ps, PPs and EMPs, respectively (*p* = 0.013). Patients with PPs who underwent ASCT had similar OS than those without Ps (98 vs. 113 months) and significantly longer than those with EMPs (98 vs 47 months, *p* = 0.006). In patients non-eligible for ASCT the presence of PPs or EMPs was associated with shorter OS compared with patients without Ps (32 vs. 24 vs. 6 months, *p* = 0.009). In the relapsed setting, a significant survival benefit was observed beyond the year 2000, but still with significant differences among patients without Ps, PPs and EMPs (37 vs 22 vs 16 months, *p* = 0.003). Importantly, rescue therapy with combinations of proteasome-inhibitors plus immunomodulatory drugs was associated with prolonged OS from first relapse (over 6 years), even in patients with EMPs.

## Introduction

Multiple myeloma (MM) is a proliferation of clonal plasma cells that produce a monoclonal protein detectable in serum and/or urine and is characterized by the presence of CRAB manifestations (hypercalcemia, renal failure, anemia, bone disease). Although myeloma plasma cells have a strong dependence of the bone marrow microenvironment, in up to one-third of patients the plasma cell proliferation escape the microenvironment influences resulting in soft-tissue plasmacytomas [1–4]. In fact, between 15-20% of patients present plasmacytomas at diagnosis and ~30% will develop plasmacytomas at relapse. However, despite its high frequency, many aspects of extramedullary disease (EMD) remain unknown. This fact is partially explained by the lack of uniform definition of EMD in the literature. Thus, while some authors consider only soft-tissue plasmacytomas as EMD, other authors include also plasmacytomas arising from bone. In an attempt to clarify and unify the nomenclature, a consensus report has recently been published that classifies plasmacytomas into two types based on their origin: (1) paraskeletal plasmacytomas (PPs), consisting of soft-tissue masses that arise from focal bone lesions and disrupt the cortical bone and (2) extramedullary plasmacytomas (EMPs), consisting of soft-tissue masses with no contact with bone as a result of hematogenous spread [5]. Rarely, plasmacytomas can arise from sites that previously suffered traumatic injuries, such as surgical scars, catheter insertions or bone fractures [6–8].

The presence of plasmacytomas is considered an adverse prognostic factor, being worse for EMPs in comparison with PPs [2, 9–11]. Although the overall survival (OS) of patients with MM has improved substantially since the introduction of novel agents, the treatment of EMD remains challenging [12]. In fact, in the Arkansas experience, the survival of patients with EMD was significantly shorter, even if they received therapy with novel agents [13]. Treatment recommendations are difficult to establish because of the lack of data coming from large series and are often based on expert opinions or retrospective analysis. In addition, and although not reported in the literature, the presence of plasmacytomas is often associated with a worse quality of life and the need of adjunctive therapy such as local radiation therapy.

In this context, the aim of our study was to analyze the incidence, location and outcome of patients with MM and plasmacytomas diagnosed at our institution over a long period of time, focusing on the two types of plasmacytomas: PPs and EMPs

## Patients And Methods

### Patients

All patients with MM diagnosed and treated at Hospital Clínic from Barcelona (Catalonia-Spain) between January 1970 and December 2018 were included in the study. Patients with primary plasma cell leukemia or solitary plasmacytoma were excluded. Baseline characteristics, treatment and follow-up data were obtained from our database continuosly updated. Plasmacytomas were assessed by physical examination and/or imaging methods and histologically confirmed whenever consider necessary. Plasmacytomas arising from a lytic bone lesion were classified as PPs, while plasmacytomas with no contact with bone were considered EMPs. Patients presenting simultaneously with both, PPs and EMPs, were considered as EMPs for the survival analysis. Medical records and radiological studies were accurately reviewed to determine the locations and type of plasmacytomas. All data were reviewed by at least one senior author of the paper (JB or LR). Cytogenetic data were available in a minority of patients for historical reasons and were not considered in the analysis. The International Staging System (ISS) was retrospectively applied in all patients with albumin and β2-microglobulin measurements at diagnosis. Patients were categorized into two calendar periods according to the availability of novel agents: 1970-1999 (period 1) and 2000-2018 (period 2). Therapeutic regimens were grouped in 5 categories: conventional chemotherapy-based, proteasome inhibitor-based (PI), immunomodulatory-based (IMiD), proteasome inhibitor plus immunomodulatory drug based (PI + IMiD) and monoclonal antibody-based (MAb). Response to therapy and progression were assessed according to Uniform Response Criteria for MM [14]. This study was conducted with the approval of the Hospital Clínic of Barcelona Institutional Review Board and in accordance with the Declaration of Helsinki. Informed consent was obtained from all subjects.

### Statistical methods

Categorical variables were described as frequency and percentage and continuous variables were reported as mean and standard deviation (SD) or median and range. The χ^2^ or Fisher’s exact test was used when required to assess the statistical significance of multiple comparisons. Overall survival (OS) was calculated from the date of diagnosis to death or last follow-up for censored cases. Survival curves were plotted according to the Kaplan and Meier method and statistically compared by means of the log-rank test.

All analyses and graphs were obtained using the statistical software IBM SPSS version 25.

## Results

### Characteristics of patients at diagnosis

A total of 1304 patients were included in the analysis and 256 of them (19.6%) had plasmacytomas at diagnosis. Patient’s baseline characteristics are summarized in Table 1. Median age of patients with and without plasmacytomas was 61 years (range 24–87) and 65 (range 21–92), respectively. There was a predominance of male gender in the group with plasmacytomas (57.8% vs. 50.1%, *p* = 0.03). Patients with plasmacytomas at diagnosis had a higher percentage of Bence-Jones (19.5% vs. 12.4%, *p* = 0.004) and oligosecretory myeloma (3.1% vs.0.4%, *p* = 0.0005), as well as a less advanced ISS stage (ISS I: 49.7% vs. 25.7%, ISS 2: 27% vs. 34.3%, ISS 3: 23.2% vs. 39.9% in patients with and without plasmacytomas, respectively). Of note, the serum M-protein size (24.5 g/L vs 35.3 g/L, *p* = 0.0001) and bone marrow infiltratrion (31% vs 50%, *p* = 0.001) was significantly lower in patients with PPs compared with patients without plasmacytomas.

**Table 1:** Baseline characteristics of the patients.

	Overall series *N* = 1304	Pts without Ps *N* = 1048	Pts with Ps *N* = 256	*P*-value
Gender (male), *n* (%)	674 (51.6%)	526 (50.15)	148 (57.8%)	0.03
Age (years), median (range)	64 (21-92)	65 (21-92)	61 (24-87)	
ISS, *n* (%)*				
• I	266 (30.8%)	174 (25.7%)	92 (49.7%)	<0.0001
• II	282 (32.7%)	232 (34.3%)	50 (27%)	0.06
• III	313 (36.3%)	270 (39.9%)	43 (23.2%)	<0.0001
Heavy chain type, *n* (%)				
• IgG	703 (53.9%)	579 (55.2%)	124 (48.4%)	
• IgA	360 (27.6%)	295 (28.1%)	65 (25.3%)	
• Ligh chain	180 (13.8%)	130 (12.4%)	50 (19.5%)	0.004
• IgD	21 (1.6%)	17 (1.6%)	4 (1.5%)	NS
• IgM	8 (0.6%)	6 (0.5%)	2 (0.7%)	NS
• Oligosecretory	13 (0.9%)	5 (0.4%)	8 (3.1%)	0.0005
• Biclonal	12 (0.9%)	10 (0.9%9)	2 (0.7%)	NS
• Unknown	7 (0.5%9)	6 (0.5%)	1 (0.3%)	NS
Ligh chain type, *n* (%)				
• Kappa	722 (55.3%)	582 (55.5%)	140 (54.6%)	NS
• Lambda	530 (40.6%)	427 (40.7%9)	103 (40.2%)	
• Non-secretory	14 (1.07%)	6 (0.5%)	8 (3.1%)	0.001
• Biclonal	9 (0.69%)	7 (0.6%)	2 (0.7%)	NS
• Unknown	29 (2.2%)	26 (2.4%)	3 (1.1%)	
Serum M-protein (g/L)(mean ± SD)	33.2 ± 21.9	35.3 ± 21.6	24.5 ± 21.08	0.0001
Bone marrow plasma cells (%)(mean ± SD)	46 ± 28.9	50 ± 27.8	31 ± 28.8	0.0001

^*^Available in 861 patients.

### Incidence and location of Ps at diagnosis

The incidence of the two types of plasmacytomas at diagnosis was 17.6% (230 patients) and 1.9% (26 patients) for PPs and EMPs, respectively. There were not significant differences in baseline characteristics between patients with both subtypes of plasmacytomas (Supplementary Table 1).

Overall, the incidence of soft-tissue plasmacytomas increased overtime, from 15.4% in period 1 to 22.9% in period 2, being the increase more pronounced in the group of PPs (13.8% to 20.6%) than for the EMPs group (1.5% to 2.3%) (Table 2).

**Table 2:** Incidence of plasmacytomas overtime at diagnosis and at first relapse.

Plasmacytomas	Overall	Period 1	Period 2
At diagnosis	*N* = 1304	*N* = 577	*N* = 727
No	1048 (80.3%)	488 (84.5%)	560 (77%)
Yes	256 (19.6%)	89 (15.4%)	167 (22.9%)
• EMPs	26 (1.9%)	9 (1.5%)	17 (2.3%)
• PPs	230 (17.6%)	80 (13.8%)	150 (20.6%)
Relapsed patients (with data available)	*N* = 967	*N* = 415	*N* = 552
No	775 (80.1%)	330 (79.5%)	445 (79.6%)
Yes	192 (19.8%)	85 (20.4%)	107 (19.3%)
• EMPs	50 (5.1%)	19 (4.5%)	31 (5.6%)
• PPs	142 (14.6%)	66 (15.9%)	76 (13.7%)

Sixty six percent of patients presented with only one involved site while 34% had plasmacytomas in two or more locations. Within the PPs group, the most commonly involved sites were chest (40%), paravertebral (39.1%), skull (13%) and pelvis (11.3%). In the EMPs group the most commonly involved sites were pleura (23%), skin (19.2%) and liver (15.3%). Central nervous system (CNS) involvement at diagnosis was extremely uncommon with only one patient. Location of plasmacytomas is depicted in Table 3.

**Table 3:** Location of plasmacytomas at diagnosis and at first relapse.

Location*	At diagnosis	At first relapse
Paraskeletal	*N* = 230	*N* = 142
• Chest	92 (40%)	65 (45.7%)
• Paravertebral	90 (39.1%)	71 (50%)
• Skull	30 (13%)	24 (16.9%)
• Pelvis	26 (11.3%)	16 (11.2%)
• Long bones	3 (1.3%)	9 (6.3%)
Extramedullary	*N* = 26	*N* = 50
• Pleura, lung	6 (23%)	13 (26%)
• Skin, subcutaneous cell tissue, muscle	5 (19.2%)	20 (40%)
• Liver	4 (15.3%)	8 (16%)
• Other locations (EMPs: kidney, peritoneum)	15 (57.6%)	13 (26%)
• Central Nervous System	1 (3.8%)	4 (8%)

^*^34% and 56% of patients had more than one location at diagnosis and first relapse, respetively.

### First line treatment

The initial treatment evolved overtime. Thus, in the overall series, all patients diagnosed before the year 2000 were treated with chemotherapy while after the year 2000, 42.3% received chemotherapy, 24.7% PI, 9.6% IMiDs, 20.6% PI + IMiD and 2.1% MAb. Seventy-seven (13.3%) patients received an autologous stem cell transplant (ASCT) in period 1 compared with 336 (46.2%) patients in period 2. The induction treatment in each subgroup of patients according to the type of plasmacytomas is summarized in Supplementary Table 2. Overall, 21.8% of the patients receiving initial chemotherapy underwent up-front ASCT (13.6% in period 1 and 37% in period 2), compared with 44%, 28% and 80.6% of the patients receiving PI, IMiD and PI + IMiD-based regimens, respectively. Obviously, this reflects the active clinical trials and clinical guidelines overtime at our institution. Initial treatments are summarized in Table 4 and Supplementary Table 3. Eighty-three patients with plasmacytomas (32.4%) received also local radiation therapy.

**Table 4:** Treatment received at diagnosis and at first relapse in the overall series and by periods of time.

Plasmacytomas	Overall	Period 1	Period 2
At diagnosis	*N* = 1304	*N* = 577	*N* = 727
QT	873 (66.9%)	565 (97.9%)	308 (42.3%)
PI	180 (13.8%)	−	180 (24.7%)
IMiD	70 (5.3%)	−	70 (9.6%)
PI + IMiD	150 (11.5%)	−	150 (20.6%)
MAb	16 (1.2%)	−	16 (2.1%)
ASCT	413 (31.6%)	77 (13.3%)	336 (46.2%)
Relapsed patients*	*N* = 768	*N* = 305	*N* = 463
QT	386 (50.2%)	271 (88.8%)	124 (26.7%)
PI	155 (20.9%)	7 (2.2%)	154 (33.2%)
IMiD	110 (14.3%)	18 (5.9%)	92 (19.8%)
PI + IMiD	55 (7.1%)	−	55 (11.8%)
MAb	25 (3.2%)	−	25 (5.3%)

^*^Relapsed patients who received salvage therapy.

### Survival of patients with and without plasmacytomas at diagnosis

**Fig. 1:**
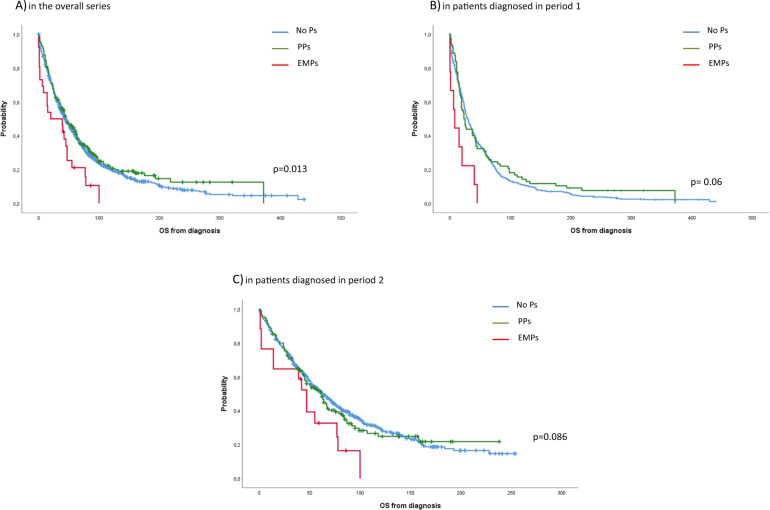
Overall survival from diagnosis. **A** in the overall series, **B** in patients diagnosed in period 1, **C** in patients diagnosed in period 2.1.

**Fig. 2:**
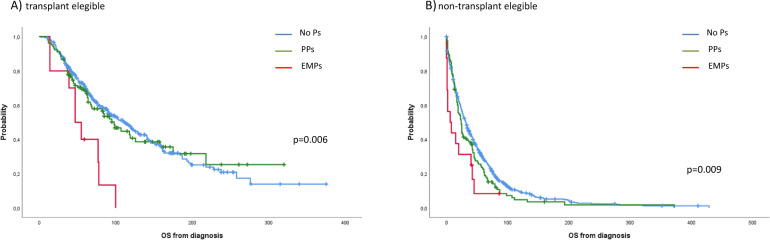
Overall survival from diagnosis. **A** In transplant eligible and **B** non-transplant eligible patients.

Overall, 276 of 1304 patients (21%) were alive at the time of this analysis with a median follow-up in survivors of 82 months. The median OS was 45, 44 and 20 months for patients without plasmacytomas at diagnosis, patients with PPs and patients with EMPs, respectively (*p* = 0.013) (Fig. 1A). Although survival outcomes improved overtime in all subgroups, patients with EMPs continued to have significantly shorter OS compared with patients with PPs and those without plasmacytomas (period 1: 8 vs. 23 vs. 29 months, *p* = 0.006; period 2: 47 vs. 62 vs. 63 months, *p* = 0.086) (Fig. 1B, C). When we analyzed the survival of patients who underwent ASCT, patients with PPs who received ASCT had similar OS than patients without plasmacytomas (median: 98 vs 113 months, *p* = 0.807) and had a significantly longer OS than patients with EMPs (98 vs 47 months, *p* = 0.006). In contrast, in patients non-transplant eligible the presence of EMD, either PPs or EMPs, confers worse prognosis compared with patients without plasmacytomas, being dismal for those with EMPs (32 vs. 24 vs. 6 months, *p* = 0.009, for patients without plasmacytomas, patients with PPs and patients with EMPs, respectively) (Fig. 2A, B). Finally, we also analyzed the impact of the initial therapy in the 3 groups of patients. Overall, patients with PPs had the same OS than patients without plasmacytomas while patients with EMPs had a shorter OS when treated with conventional chemotherapy (34 vs. 29 vs. 15 months, *p* = 0.041 for patients without plasmacytomas, PPs and EMPs, respectively). There was a trend towards a shorter OS for patients with EMD when treated with IMiD therapy (67 vs. 47 vs. 14 months, *p* = 0.07) and PI + IMiD therapy (94 vs. not reached vs. 39 months, *p* = 0.069). Patients who received a initial PI regimen had a similar OS regardless the presence of extramedullary involvement, either PPs or EMPs (67 vs. 51 vs. 47 months, *p* = 0.29) (Supplementary Fig. 1).

### Incidence and location of plasmacytomas at first relapse

Overall, 991 out of 1304 (75.9%) patients relapsed. Data regarding the presence, type and location of plasmacytomas were available in 967 of the 991 patients. One hundred ninety two (19.8%) patients developed plasmacytomas at first relapse. The incidence of PPs at first relapse was similar (14.6%) to that observed at diagnosis while the incidence of EMPs significantly increased at relapse (from 1.9% to 5.1%, *p* = 0.0046). No differences in the incidence of plasmacytomas at first relapse were observed between periods 1 and 2 (Table 2). Of interest, the incidence of plasmacytomas at first relapse in patients with and without plasmacytomas at diagnosis was 46% versus 13%, respectively (*p* < 0.00001). The incidence of plamacytomas at relapse in patients who underwent up-front ASCT vs. those who did not was 22% vs 17.8% (*p* = 0.22). No differences in the incidence of plasmacytomas were observed between patients initially treated with chemotherapy or new drugs (18.6% vs 19.4%, *p* = 0.37). Fifty six percent of patients relapsing with plasmacytomas had involvement in two or more locations. The location of PPs were similar to that observed at diagnosis (paravertebral 50%, chest 45.7%, skull 16.9%, pelvis 11.2%, long bones 6.3%). Regarding EMPs, we observed an increased incidence of skin involvement (40%), while the incidence in other locations was quite similar to that observed at diagnosis (pleura 26%, liver 16%). CNS involvement was also uncommon with only 4 patients, representing the 2% of the overall series (Table 3).

### Treatment at first relapse

Treatment of patients at first relapse is summarized in Table 4 and Supplementary Table 4. The treatment most commonly used was conventional chemotherapy (49.7%) followed by PI-based (20.9%) and IMiD-based (13.9%). As in the first line, treatment at relapse evolved overtime. Thus, in period 2 only 26% of the patients were rescued with chemotherapy while the remaining received salvage therapy with new drugs. Overall, salvage therapy was similar in patients with and without plasmacytomas, although IMiD-based regimens were more frequently used in patients without plasmacytomas (16.3% vs 8.9%, *p* = 0.01).

### Survival of patients with and without plasmacytomas at first relapse

**Fig. 3:**
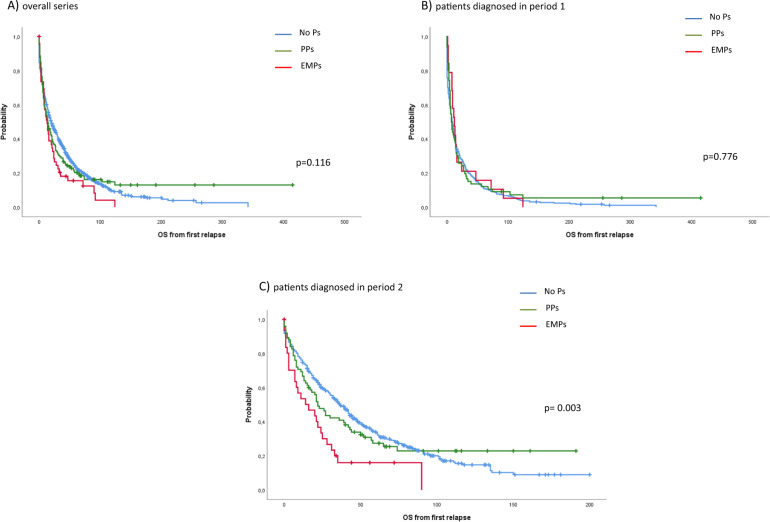
Overall survival from first relapse. **A** Overall series, **B** patients diagnosed in period 1, and **C** patients diagnosed in period 2.

In the overall series, the OS from the time of first relapse was not significantly different among patients who relapsed without plasmacytomas compared with those who developed PPs or EMPs (20 vs. 14 vs. 13 months, *p* = 0.116) (Fig. 3A). We compared the outcome from first relapse in patients diagnosed before or after the availability of new drugs (year 2000). The outcome from first relapse in patients diagnosed in period 1 was dismal, with a median OS of 8 vs. 7 vs. 12 months (*p* = 0.776) for patients without plasmacytomas, patients with PPs or patients with EMPs (Fig. 3B). In patients diagnosed in period 2, the OS from first relapse significantly improved in patients relapsing without plasmacytomas, while the improvement in patients with plasmacytomas was limited, particularly in those relapsing with EMPs (37 vs. 22 vs. 16 months, *p* = 0.003, respectively) (Fig. 3C). The OS from first relapse in patients receiving salvage therapy with chemotherapy was dismal, with a significantly longer OS in patients without plasmacytomas (17 vs. 8 vs. 9 months, for patients without plasmacytomas, patients with PPs and patients with EMPs, respectively, *p* = 0.006). PI or IMiD-based regimens improved the outcome in all subgroups, although the benefit was lower for patients with plasmacytomas. Thus, in patients receiving a PI-based regimen the OS was 53, 22 and 16 months (*p* = 0.0001) for patients without plasmacytomas, with PPs and with EMPs, respectively. In patients receiving an IMiD-based regimen the OS was 49, 34 and 25 months (*p* = 0.117), respectively. Salvage therapy with a PI + IMiD based-regimen substantially improved the median OS in all subgroups of patients with no statistically significant difference among them: 73 vs. not reached vs. 90 months (*p* = 0.414) for patients without plasmacytomas, patients with PPs and patients with EMPs, respectively (Supplementary Fig. 2). IP + IMiD based-regimen consisted of KRd (carfilzomib, lenalidomide and dexamethasone) in most cases (Supplementary Table 4).

## Discussion

This study describes the incidence, presenting features and impact on survival of EMD in a large cohort of patients diagnosed with MM and followed at an academic institution over a long period of time, from 1970 to 2018. According to the consensus on EMD definition recently published [5], the outcome of the two types of plasmacytomas, PPs and EMPs, were analyzed separately. The incidence at diagnosis was 17.6% and 1.9% for PPs and EMPs, respectively. Nevertheless, the incidence of plasmacytomas has increased from 15.4% to 22.9% in recent years, a fact likely due to the wider use of more sensitive imaging techniques such as CT scan, MRI or PET/CT [15–17]. In fact, this increase has been particularly observed in PPs, as they often originate in locations adjacent to bones that may be clinically silent. At relapse, the incidence of PPs was similar than at diagnosis (14.6%) but the incidence of EMPs increased up to 5.1%. This observation is in line with the reported incidence in a recent meta-analysis [12]. Thus, at diagnosis, the reported incidence ranges from 7% to 34.4% [1–4, 9, 11] for PPs and from 1.75% to 4.5% [3, 4, 13, 18] for EMPs. At relapse, the incidence of PPs remains similar (6% to 34.2%) [3, 4, 9, 19, 20] while the incidence of EMPs increases from 3.4% up to 10% [1–4, 9, 13, 18, 19]. The critical risk factor for the development of plasmacytomas at relapse is the presence of plasmacytomas at diagnosis. We found that 46% of patients with plasmacytomas at diagnosis developed plasmacytomas at first relapse compared to only 13% in those without initial soft-tissue involvement. Interestingly, the risk did not increase by previous therapy with novel agents, mainly thalidomide, or ASCT. In a large Italian study [2] including 1003 patients with MM the presence of plasmacytomas at diagnosis was the only significant risk factor for recurrence of plasmacytomas at relapse and, in accordance with our study, the risk did not not increase by prior exposure to novel agents or ASCT. In addition, a report from de Dana Farber Cancer Institute showed no increase in the risk of development of extramedullary disease (EMPs or PPs) in patients with de novo MM treated upfront with bortezomib/lenalidomide combinations [20] or who received ASCT [18]. These findings definitively support that the characteristics of the plasma cell clone itself rather that the type of treatment received is responsible for the soft-tissue growth and spread in MM [5]. Some of these findings have been observed also by others [2, 9, 10, 21, 22]. Of interest, we found that patients with plasmacytomas had a significantly lower serum M-protein size and a significantly lower BMPC infiltration than those without plasmacytomas. Lee et al. also reported that patients with plasmacytomas showed significantly lower levels of serum M-protein in a smaller series [10]. In this sense, the better prognostic features associated with the presence of plasmacytomas, such as the more favorable ISS, lower serum M-protein size and lower BMPC infiltration might reflect differences in the disease biology that could also explain why these patients develop plasmacytomas more frequently at relapse. In fact, an increased prevalence of high-risk cytogenetics, particularly del17p13 or TP53 mutations has been described in patients with EMD, both at diagnosis and at relapse [23, 24]. Moreover, some studies have found a higher frequency of TP53 mutations or high-risk cytogenetics abnormalities in extramedullary sites compared with paired bone marrow samples [25, 26]. In the Arkansas experience [13], EMD was associated with high risk features by gene expression profile analysis and interestingly, no different recurrent cytogenetic abnormalities have been found between the two types of plasmacytomas [27].

Extramedullary disease is generally considered a poor prognostic feature, but few reports discriminate between the two types of plasmacytomas. Wu et al. reported a shorter survival for patients with de novo MM and plasmacytomas treated with conventional chemotherapy compared with patients without plasmacytomas. However, patients who underwent ASCT had similar outcome, irrespective of the presence or absence of plasmacytomas [9]. Varetoni et al. described a series of 76 out of 1003 patients with plasmacytomas at diagnosis treated with conventional chemotherapy with a shorter PFS (18 vs. 30 months) compared with patients without plasmacytomas while the median OS was not statistically different between the two groups. In this study, patients with plasmacytomas who received ASCT had a similar PFS and OS than patients without plasmacytomas [2]. More recently, in the era of new drugs, Lee et al. reported a shorter PFS and OS in patients with initial plasmacytomas. This adverse prognostic impact of plasmacytomas was only observed in transplant-ineligible patients, and it was attenuated when bortezomib was administered [10]. Usmani et al. reported a shorter PFS and OS in patients with extramedullary disease, regardless if they were treated in Total Therapy (TT) protocols, non-TT protocols or non-protocol therapy [13]. A recent metanalysis including 2332 patients with newly diagnosed MM enrolled in 8 clinical trials for transplant-eligible and non-transplant eligible patients has been reported. Overall, 267 patients (11.4%) had soft-tissue masses, being paraskeletal in 243 (10.4%) and extramedullary in 12 (0.5%). All patients were treated with IMiDs, mainly lenalidomide, and/or PIs and there was no significant difference in PFS between patients with and without plasmacytomas (25.3 vs. 25.2 months). However, the presence of EMD was associated with a shorter OS (63.5 vs. 79.9 months, *p* = 0.01) [21]. This is in line with the results of the Spanish trial GEM05menos65, showing no significant difference in PFS between patients with or without plasmacytomas (paraskeletal in almost all cases) but significantly shorter OS in those with plasmacytomas [28]. Two studies separately analyzed the outcome of patients with PPs and EMPs [11, 29]. A large retrospective study of the European Society for Blood and Marrow Transplantation (EBMT) registry included 3744 patients with newly diagnosed MM who received up-front ASCT, and the incidence of PPs and EMPs were 14.5% and 3.7%, respectively. Of interest, no different outcomes were observed between patients with PPs and patients without plasmacytomas (3-yr PFS 47.9% vs. 50%, *p* = 0.78 and 3-yr OS 80.1% vs. 77.7%, *p* = 0.09). In contrast, patients with EMPs had a significantly worse 3-yr PFS of 39.9% in comparison to patients without plasmacytomas (*p* = 0.001) and PPs patients (*p* = 0.007), and a significantly worse 3-yr OS of 58% compared to patients without plasmacytomas and patients with PPs (*p* < 0.001 in both cases) [11]. In a retrospective study of 130 patients presenting with extramedullary or paraskeletal involvement, the median PFS was 38.9 vs. 51.7 months (*p* = 0.034) and the median OS was 46.5 vs. not reached (*p* = 0.002) for the EMPs and PPs groups, respectively [29]. Our results show that patients with PPs display similar outcomes than patients without plasmacytomas and a significantly longer OS than patients with EMPs. Of interest, despite the introduction of new drugs and the resulting survival improvement in the overall series, the worse prognosis associated with EMPs is retained in period 2 although without statistical significance (63 vs. 62 vs. 47 months, *P* = 0.086 for patients without plasmacytomas, patients with PPs and patients with EMPs, respectively). As reported by others, ASCT overcomes the bad prognosis of PPs, but not EMPs [2, 9–11, 29]. Thus, in our series, the median OS of patients with PPs is >8 years compared to 4 years in patients with EMPs. In non-transplant eligible patients, the median survival of patients with EMPs at diagnosis was only 6 months. No conclusions can be drawn about the efficacy of different types of treatments over EMPs, given the low number of patients in our series and the retrospective nature of the study. However, our results suggest that patients with EMPs still have inferior outcomes despite the survival improvement achieved with the introduction of novel agents.

In the relapse setting, Pour et al. reported that soft-tissue involvement was associated with a poor prognosis, particularly in patients with EMPs compared with PPs (median OS from relapse of 5 vs. 12 months, *p* = 0.006) [19]. Mangiacavalli et al. also reported a short survival for patients relapsing with plasmacytomas, being worse for those with EMPs compared with PPs (1.6 vs. 2.4 years, *p* = 0.006) [30]. More recently, Beksac et al. also reported the different outcome of the two types of soft-tissue involvement. Thus, patients relapsing with EMPs had a shorter OS than patients with PPs (11.4 vs. 39.8 months, *p* = 0.093) [29]. According to our experience, the survival after first relapse is dismal in all subgroups of patients, regardless of the presence or not of plasmacytomas. However, outcomes have significantly improved beyond the year 2000, with a median OS of 37, 22 and 16 months for patients without plasmacytomas, with PPs and with EMPs, respectively. Of note, the introduction of new drugs has meant an important step forward, with the major benefit observed in patients receiving a PI + IMiD rescue therapy, resulting in a median OS from first relapse over 6 years. Although these advances have been observed in all groups of patients, including those with EMPs, there is still a significant room for improvement. Unfortunately, daratumumab [31, 32] and the XPO-1 inhibitor selinexor [33] have shown limited efficacy in patients with advanced disease and plasmacytomas. The peptidase conjugate melflufen plus dexamethasone resulted in an encouraging 25% and 22% responses in patients with PPs and EMPs, respectively [34]. With the limitations of the standard approaches newer immunotherapies, such as toxin immunoconjugate MoAbs, bispecific antibodies and the CAR-T cell approach are the most promising. Belantamab mafodotin, a conjugated MoAb against BCMA has shown limited efficacy with only 7.5% responses in patients with advanced myeloma and plasmacytomas [35]. In a recent study, 28 out of 165 patients treated with the T-cell-redirecting bispecific antibody teclistamab had plasmacytomas and a lower response rate was observed in this population [36]. There are no survival data with bispecific antibodies in patients with plasmacytomas. Concerning the CAR-T cell approach, the response rate seems to be similar to that observed in patients without plasmacytomas with quick disappearance of soft-tissue involvement [37, 38]. Thus, in one study eight of nine patients with plasmacytomas responded to CAR-T, including 4 CRs and 2 VGPR [37]. However, in two studies patients with EMD have shown unsatisfactory long-term outcome with shorter PFS and OS than those with no EMD [39, 40]. This is worrisome and need to be further explored in forthcoming studies. Finally, the adoption of response criteria based on both morphological and functional evaluation, such as the standardization of 18F-FDG-PET/CT as recently proposed by Zamagni et al. [41] will hopefully help to more accurately assess the presence and the response to therapy of EMD.

In summary, patients with plasmacytomas have less tumor burden than those without plasmacytomas. It is important to distinguish between the two types of plasmacytomas, as they display different outcomes. EMPs are more frequent at relapse than at diagnosis while the incidence of PPs is similar at diagnosis and at relapse. The only factor associated with the development of plasmacytomas at relapse is the presence of plasmacytomas at diagnosis. Patients with PPs at diagnosis undergoing high-dose therapy have similar survival than those without plasmacytomas while patients with EMPs had poorer outcome than those with PPs or those without soft-tissue involvement. At relapse, there are still significant differences in survival among patients without plasmacytomas, with PPs and with EMPs, despite the improvement in outcomes observed in all subgroups after the introduction of novel drugs. Prospective analysis focused on survival outcomes in patients with MM and clearly defined PPs and EMPs with the new wave of antimyeloma approaches should be encouraged.

## Supplementary information

Supplementary tables and figures

## Data Availability

All data generated or analyzed during this study are included in this published article (and its supplementary information files).
