# Supplementary material for: Paraskeletal and extramedullary plasmacytomas in multiple myeloma at diagnosis and at first relapse: 50-years of experience from an academic institution
Source: Blood Cancer J. 2022 Sep 16;12(9):135. doi: 10.1038/s41408-022-00730-5 (PMC9481598; doi:10.1038/s41408-022-00730-5)
Supplement: Supplementary file 1 — Supplementary tables and figures [file 41408_2022_730_MOESM1_ESM.pdf]

Supplementary table 1: Baseline characterisitics of the patients with PPs and EMPs

	PPs<br>N= 230	EMPs<br>N=26	P value
**Gender (male), n (%)**	133 (57.8%)	15 (57.6%)	
**Age (years), median(range)**			
**ISS , n(%)***			
• **I**	84 (50%)	8 (47%)	NS
• **II**	47 (27.9%)	3 (17.6%)	NS
• **III**	37 (22%)	6 (35%9	NS
**Heavy chain type, n(%)**			
• **IgG**	110 (47.8%)	14 (53.8%)	NS
• **IgA**	58 (25.2%)	7 (26.9%)	NS
• **Ligh chain**	47 (20.4%)	3 (11.5%)	NS
• **IgD**	4 (1.7%)	-	NS
• **IgM**	2 (0.8%)	-	
• **Non- secretory**	7 (3%)	1 (3.8%)	NS
• **Biclonal**	2 (0.8%)	-	
• **Unknown**		1 (3.8%)	
**Ligh chain type, n (%)**			
• **Kappa**	129 (56%)	11 (42.3%9	NS
• **Lambda**	91 (39.5%)	12 (46.1%)	NS
• **Non-secretory**	7 (3%)	1 (3.8%)	NS
• **Biclonal**	2 (0.08%)	-	
• **Unknown**	1 (0.04%)	2 (7.6%)	
**Serum M-protein s (g/L)(mean±SD)**	24.05±21.3	29.07±18.2	NS
**Bone marrow plasma cells (%)(mean±SD)**	31±28	31±35	NS

*Available in 185 patients

Supplementary table S2: Initial therapy in the overall series according to the presence or absence of Ps

	Patients without Ps<br>N=1048	Patients with Ps<br>N=256	P value
**QT**	728 (69.4%)	145 (56.6%)	<0.001
**PI**	134 (12.7%)	46 (17.9%)	0.03
**IMiD**	55 (5.2%)	15 (5.8%)	NS
**PI+IMiD**	106 (10.1%)	44 (17.1%)	0.001
**MAb**	13 (1.2%)	3 (1.1%)	NS

Supplementary table 3: detailled therapy at initial diagnosis in the overall series according to the presence or absence of Ps

	Patients without Ps N=1048	Patients with Ps N=256	P value
**QT**	728 (69.4%)	145 (56.6%)	<0.001
• **Melphalan/prednisone**	285 (27.1%)	45 (17.5%)	
• **Cyclophosphamide/dexa or prednisone**	120 (11.4%)	19 (7.4%)	
• **VCMP/VBAP**	106 (10.1%)	22 (8.5%)	
• **VBMCP/VBAD**	103 (9.8%)	28 (10.9%)	
• **VAD**	26 (2.4%)	7 (2.7%)	
• **Others**	88 (8.3%)	24 (9.3%)	
**PI**	134 (12.7%)	46 (17.9%)	0.03
• **Bortezomib/dexamethasone**	67 (6.3%)	19 (7.4%)	
• **MPV**	36 (3.4%)	19 (7.4%)	
• **Others**	31 (2.9%)	8 (3.1%)	
**IMiD**	55 (5.2%)	15 (5.8%)	NS
• **Thal/dex**	20 (1.9%)	10 (3.9%)	
• **Len/dex**	16 (1.5%)	5 (1.9%)	
• **LEn/dex/clarithromicyn**	12 (1.1%)	-	
• **Others**	5 (0.4%)		
**PI+IMiD**	106 (10.1%)	44 (17.1%)	0.001
• **VTD**	71 (6.7%)	30 (11.7%)	
• **VRD**	18 (1.7%)	8 (3.1%)	
• **MPV/Rd**	10 (0.9%)	3 (1.1%)	
• **Others**	7 (0.6%)	3 (1.1%)	
**MAb**	13 (1.2%)	3 (1.1%)	NS
• **MPV-daratumumab**	7 (0.6%)	2 (0.7%)	
• **Lena/dexa/elotuzumab**	5 (0.4%)	1 (0.3%)	

Supplementary table 4: detailled therapy at first relapse in the overall series according to the presence or absence of Ps

	Patients without Ps N=775	Patients with Ps N=192	P value
**Received salvage therapy**	N= 576	N= 178	
**QT**	298	88	NS
• **Melphalan/prednisone**	66	9	
• **Cyclophosphamide/dexa or prednisone**	87	22	
• **VCMP/VBAP**	21		
• **VBAD or VBAP**	54	20	
• **Others**	70	30	
**PI**	118	40	NS
• **Bortezomib/dexamethasone**	45	15	
• **bortezomib**	23	7	
• **Carfilzomib/dexamethasone**	12	2	
• **MPV**	10	2	
• **Others**	28	14	
**IMiD**	94	16	0.01
• **Len/dex**	66	9	
• **Thalidomide**	23	5	
• **Others**	5	2	
**PI+IMiD**	38	17	NS
• **KRd**	29	14	
• **Others**	9	3	
**MAb**	25	-	
• **DRd**	10		
• **DVd**	7		
• **DKd**	6		
• **Others**	2		

Supplementary figure 1: overall survival from diagnosis according to the initial therapy a) conventional chemotherapy, b) PI-based, c) IMiD-based, d) PI+IMiD-based

A

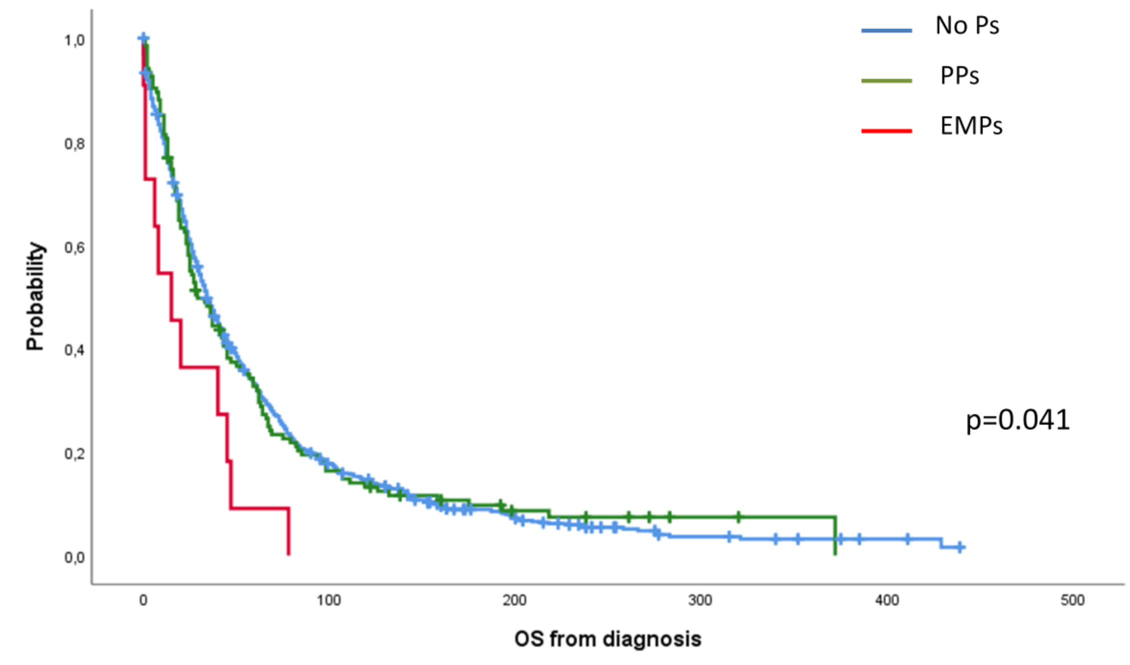

B

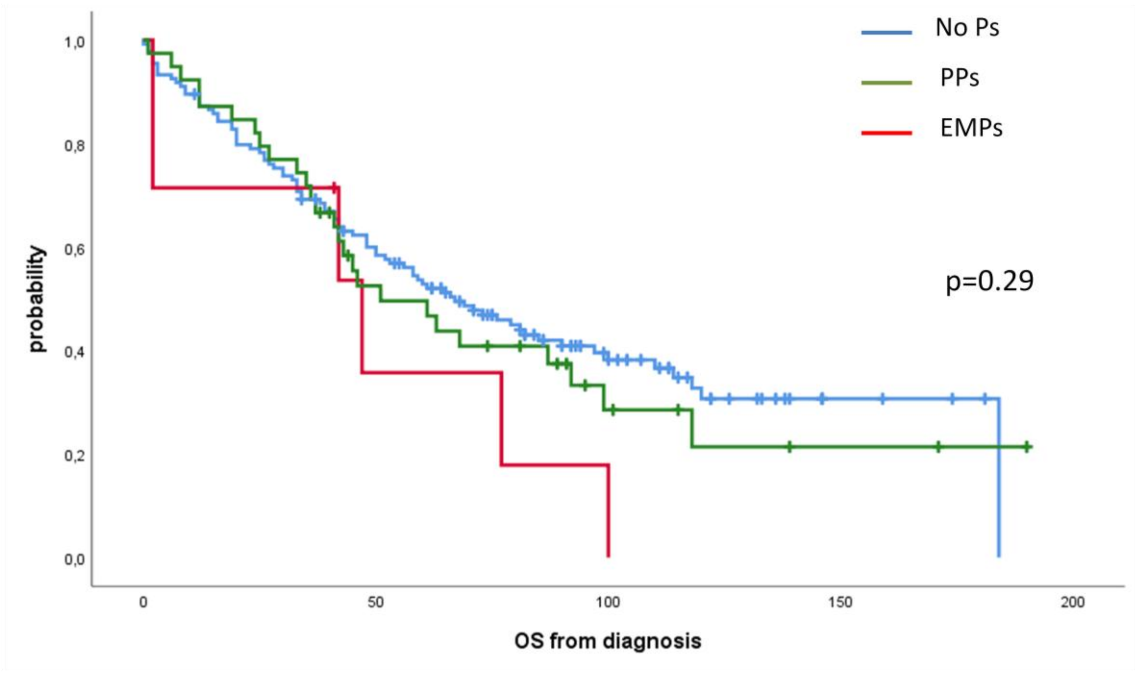

C

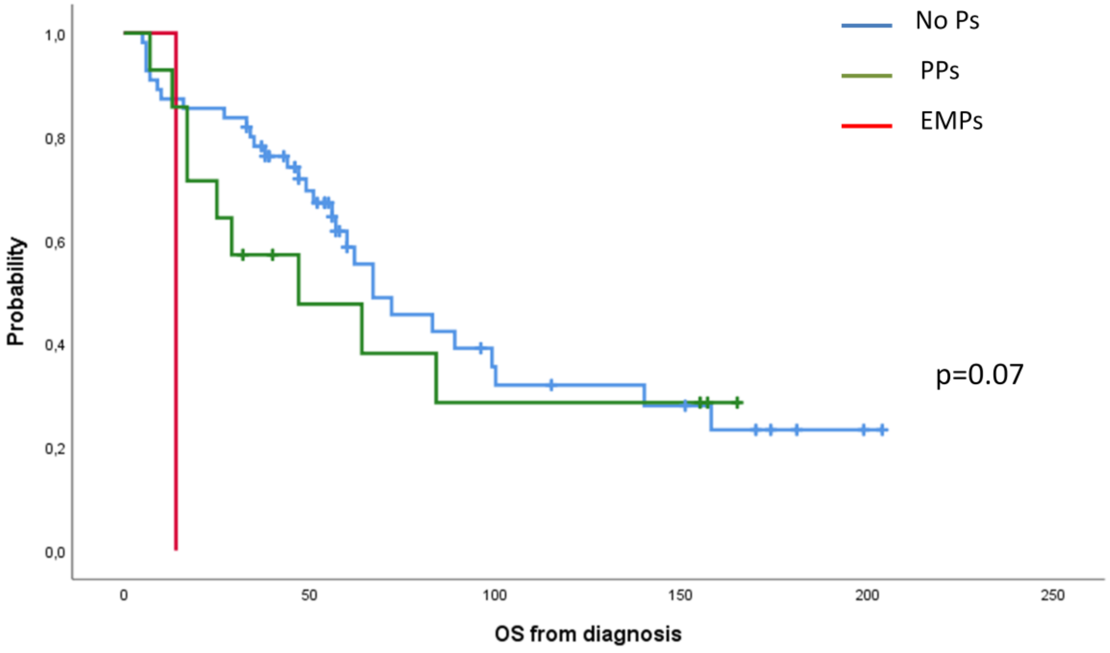
No Ps
PPs
EMPs
p=0.07
Probability
1,0
0,8
0,6
0,4
0,2
0,0
0
50
100
150
200
250
OS from diagnosis

D

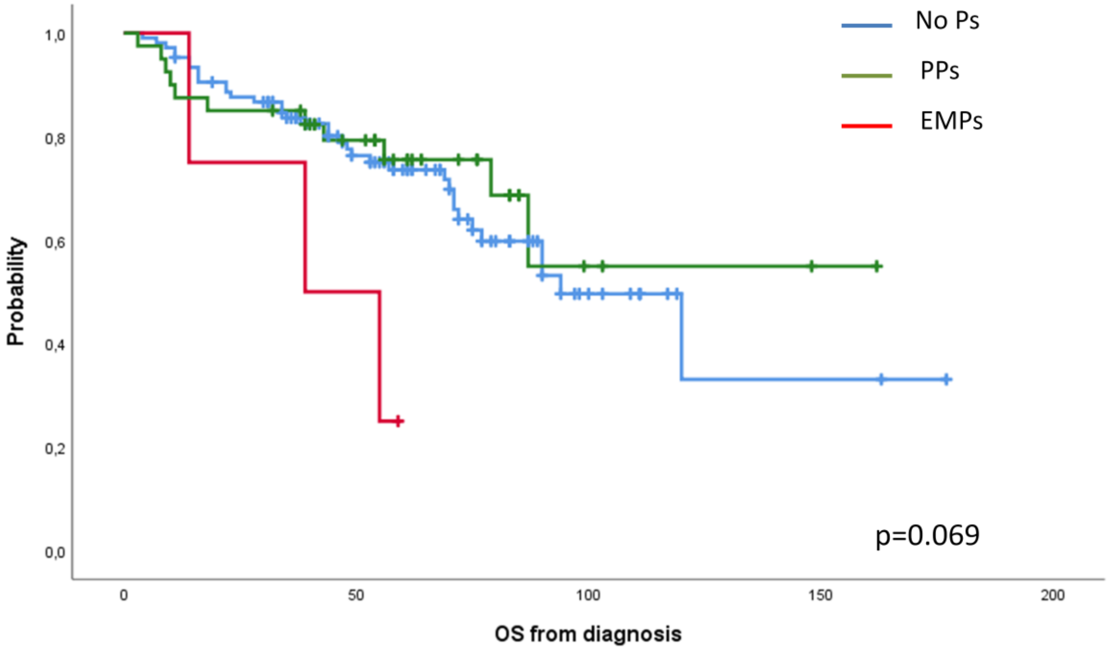
No Ps
PPs
EMPs
p=0.069
Probability
1,0
0,8
0,6
0,4
0,2
0,0
0
50
100
150
200
OS from diagnosis

Supplementary figure 2: overall survival from first relapse according to salvage therapy a) conventional chemotherapy, b) PI-based, c) IMiD-based, d) PI+IMiD-based

A

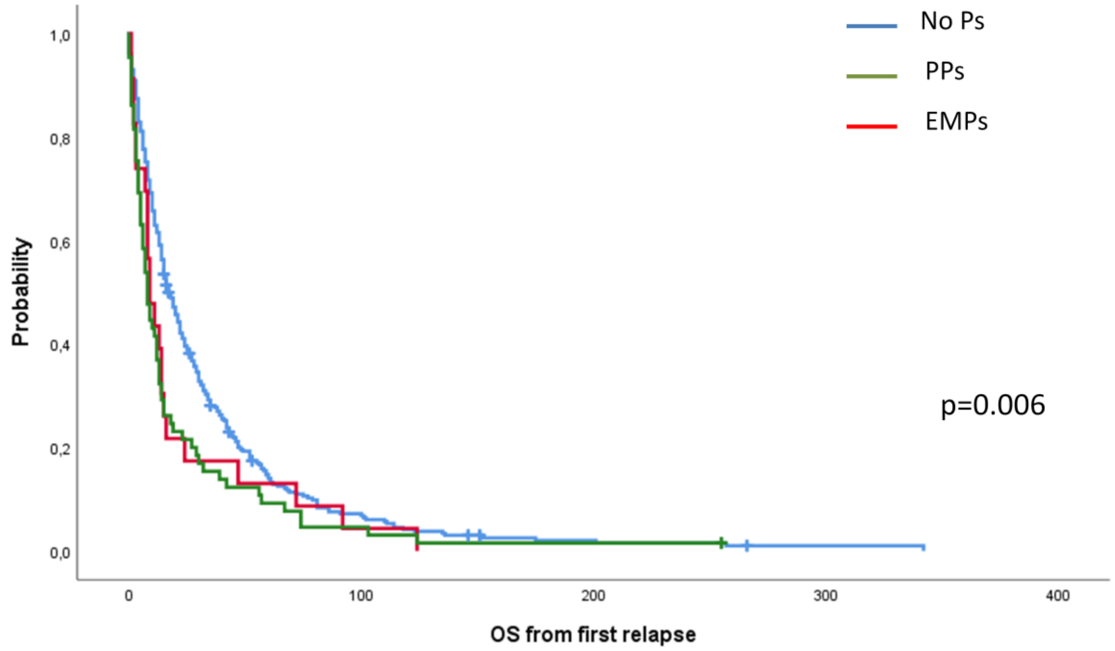

B

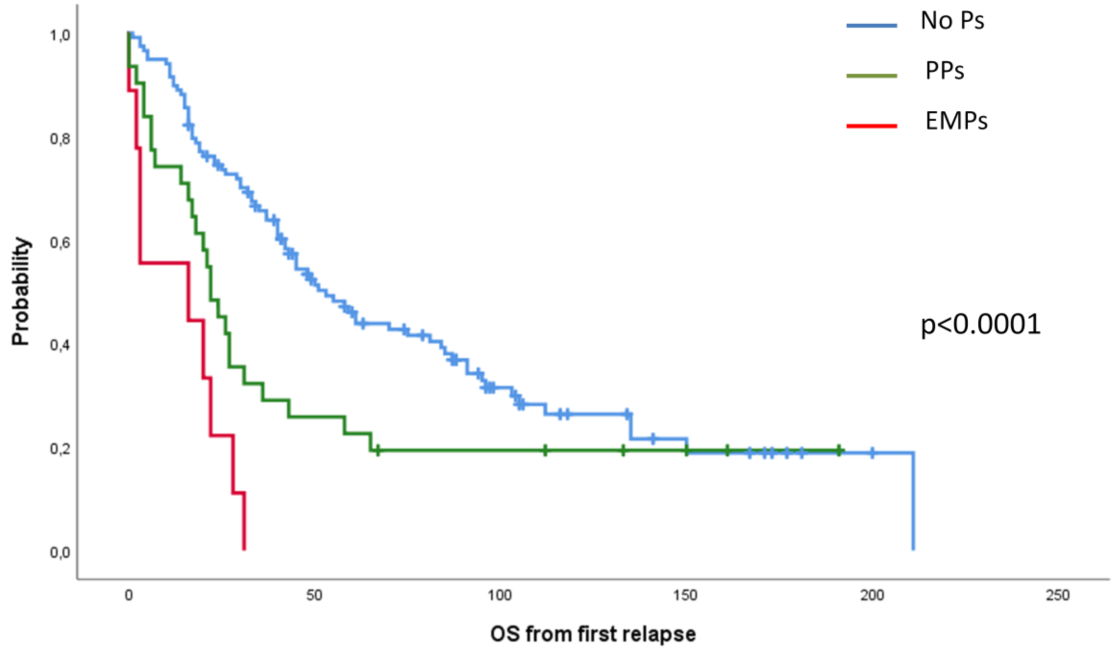

C

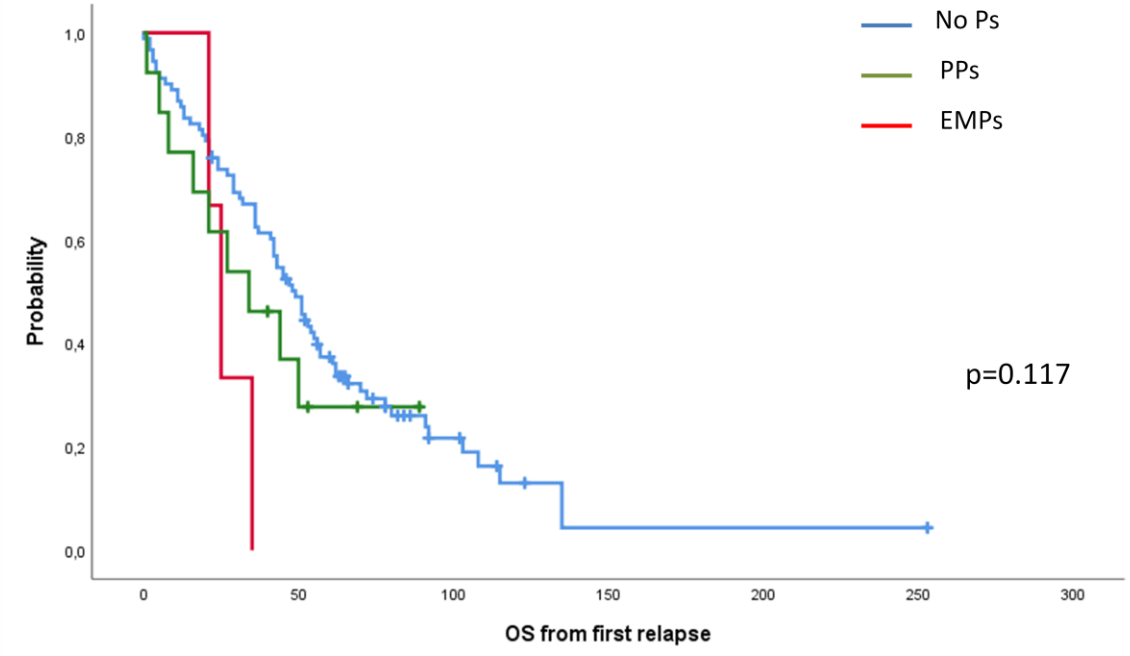

No Ps
PPs
EMPs
p=0.117
Probability
1,0
0,8
0,6
0,4
0,2
0,0
0
50
100
150
200
250
300
OS from first relapse

D

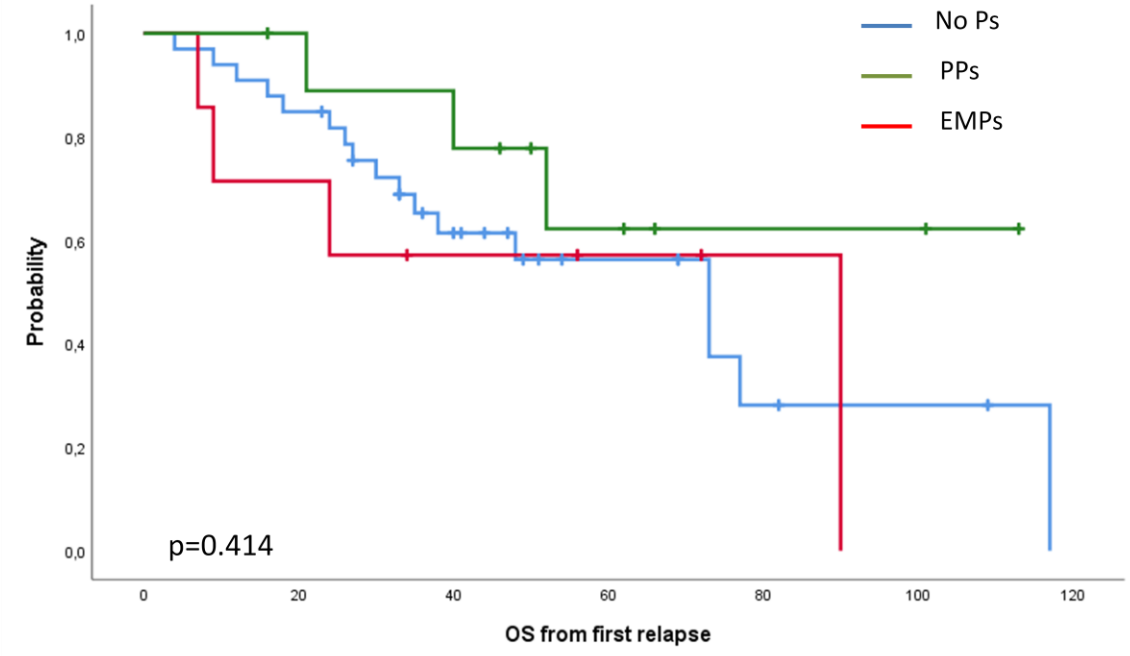

No Ps
PPs
EMPs
p=0.414
Probability
1,0
0,8
0,6
0,4
0,2
0,0
0
20
40
60
80
100
120
OS from first relapse
